# Combining backpropagation with Equilibrium Propagation to improve an Actor-Critic reinforcement learning framework

**DOI:** 10.3389/fncom.2022.980613

**Published:** 2022-08-23

**Authors:** Yoshimasa Kubo, Eric Chalmers, Artur Luczak

**Affiliations:** ^1^Canadian Centre for Behavioural Neuroscience, University of Lethbridge, Lethbridge, AB, Canada; ^2^Department of Mathematics and Computing, Mount Royal University, Calgary, AB, Canada

**Keywords:** Equilibrium Propagation, Actor-Critic (AC), biologically plausible, reinforcement learning, backpropagation

## Abstract

Backpropagation (BP) has been used to train neural networks for many years, allowing them to solve a wide variety of tasks like image classification, speech recognition, and reinforcement learning tasks. But the biological plausibility of BP as a mechanism of neural learning has been questioned. Equilibrium Propagation (EP) has been proposed as a more biologically plausible alternative and achieves comparable accuracy on the CIFAR-10 image classification task. This study proposes the first EP-based reinforcement learning architecture: an Actor-Critic architecture with the actor network trained by EP. We show that this model can solve the basic control tasks often used as benchmarks for BP-based models. Interestingly, our trained model demonstrates more consistent high-reward behavior than a comparable model trained exclusively by BP.

## Introduction

The backpropagation (BP) algorithm (Rumelhart et al., 1986) has long been the workhorse of deep neural networks, allowing their successful application to many tasks. BP-powered neural networks have enabled reinforcement learning systems to outperform humans at Go (Silver et al., 2016) and Atari games (Mnih et al., 2015). But BP has been criticized as being not biologically plausible (it seems unlikely that neurons do anything like compute partial derivatives). It has also been observed that humans still outperform deep neural networks on many tasks, like adversarial examples (Goodfellow et al., 2014), art and music. Could more biologically plausible learning mechanisms help close this gap?

In the reinforcement learning context, one biologically plausible method is the REINFORCE framework–a policy-gradient algorithm that was described in a neuroscience context by Williams (1992). The parallels between REINFORCE and biological neural learning have been discussed by Sutton and Barto (2018) and Chung (2020), and it has led to more recent developments such as the Attention-Gated Brain Propagation approach (Pozzi et al., 2020). Actor-Critic is another reinforcement learning architecture with parallels to biological learning: several studies have seen the Actor-Critic architecture as an analog of learning mechanisms in the basal ganglia (Joel et al., 2002; Takahashi et al., 2008; Sheikhnezhad Fard, 2018). Biologically plausible reinforcement learning approaches can demonstrate more human-like behavior (Chalmers and Luczak, 2022), and so may provide important insights into human learning and intelligence.

In the supervised learning context, Equilibrium Propagation (EP) has been proposed as a more biologically plausible alternative to BP (Scellier and Bengio, 2017, 2019; Ernoult et al., 2019; O’Connor et al., 2019; Laborieux et al., 2021). EP is an extension of Contrastive Hebbian Learning (Almeida, 1987; Pineda, 1987; Baldi and Pineda, 1991) that sees the neural network as a dynamical system whose steady state can be perturbed by inputs during an initial “free” phase, and then clamped by teaching signals in a second “clamped” phase, affecting learning in a biologically realistic way. EP has successfully trained algorithms to perform image classification tasks like MNIST (LeCun et al., 1998) and CIFAR10 (Krizhevsky and Hinton, 2009), and Laborieux et al. (2021) showed that convolutional networks trained by EP can achieve comparable accuracy to BP in the CIFAR10 task. A further extension of EP by Luczak et al. (2022) showed how learning might occur in a single phase–making the algorithm even more biologically plausible–while still achieving good classification accuracy.

A biologically plausible reinforcement learning approach based on EP has not yet been proposed. Here we explore an Actor-Critic architecture trained by both BP and by brain-inspired modification of EP proposed by Luczak et al. (2022). This study provides two contributions:

1. We propose the first application of EP to reinforcement learning, in the form of an Actor-Critic architecture trained by a combination of EP (Actor) and BP (Critic).
2. We demonstrate that our architecture can solve several control tasks, and that its learned behaviors are more consistently rewarding than behaviors learned using BP alone.

## Materials and methods

This section details how our Actor-Critic architecture was implemented.

### Actor-Critic architecture

Actor-Critic is a two-part architecture for reinforcement learning. The “Actor” is a model that encapsulates the learner’s policy: it observes the current state and outputs an action to execute. The “Critic” is a separate model that estimates the value of an action given a particular state. It observes the effect of each executed action, often in the form of a difference between the predicted value of the action and the value actually experienced (a “temporal difference error”). It uses the temporal difference error as a learning signal to improve its own future value estimates, and also to update the Actor to make high-value actions more likely, and low-value actions less likely.

### Actor network (trained by Equilibrium Propagation)

Equilibrium Propagation envisions a neural network as a dynamical system that learns in two phases. First is the “free phase,” in which an input is applied and the network is allowed to equilibrate. During this phase the network dynamics obey the equations:


(1) $$x_{j,t}=x_{j,t-1}+h*\left(-x_{j,t-1}+p\,\left(\Sigma_{i}\,w_{i,j}\,x_{i,t-1}+\gamma \,\Sigma_{o}\,w_{o,j}\,x_{o,t-1}+b_{j}\right)\right)$$

(2) $$x_{o,t}=x_{o,t-1}+h*\left(-x_{o,t-1}+p\,\left(\Sigma_{j}\,w_{j,o}\,x_{j,t-1}+b_{o}\right)\right)$$

where *x* is an activation, *w* is weights for each layer, *i, j*, and *o*, are indexes of input, hidden and output layer neurons, *b* is a bias. *P* is an activation function such as the sigmoid function, and is the feedback parameter. *h* is the Euler method’s time-step. Please note that for consistency with our previous work (Luczak et al., 2022) we use letter *o* for indexing output units. We hope that “*o*” will not be confused with number *0*, which is not present in our equations.

After the network has reached a free-phase steady state, the second “clamped” phase begins. During this phase the output neurons are clamped (or rather, weakly clamped or nudged) toward the target values. In conventional EP the dynamics during this phase obey the equations:


(3) $$x_{j,t}=x_{j,t-1}+h*\left(-x_{j,t-1}+p\,\left(\Sigma_{i}\,w_{i,j}\,x_{i,t-1}+\gamma \,\Sigma_{o}\,w_{o,j}\,x_{o,t-1}+b_{j}\right)\right)$$

(4) xo,t=xo,t-1+h*(-xo,t-1+p⁢(Σ⁢wj,oj⁢xj,t-1+bo)+β*(T-xo,t-1))

where *T* is a target for the classification task.

However, in a reinforcement learning setting there is no target signal *per se*; only the reward signal, which the learner must use to estimate values of particular states and actions in the environment. To accommodate this different paradigm, our Actor network modifies Eq. 4 as follows:


(5) $$x_{o,t}=x_{o,t-1}+h*(-x_{o,t-1}+p\left(\underset{j}{\Sigma }w_{j,o}x_{j,t-1}+b_{o}\right)\;\;+\beta *V*\left(a-x_{o,t-1}\right)),$$

where *a* is the action that was taken, and *V* is the estimated value of the state, as estimated by the critic network (see Supplementary Section “Dynamics for Actor” for further discussion on the forms of Eqs 2, 5). Each *x_o* is the output of a unit corresponding to a particular action. Alternatively, *V* can be replaced with a temporal-difference-style quantity to reduce variance:


(6) $$A\,\left(s\right)=r+V\,\left(s^{{}^{\prime }}\right)-V\,\left(s\right),$$

where *s* is the current state, and *s*′ is the new state (arrived at after executing *a*). Making this substitution into Eq. 5 gives the following equation for the clamped-phase dynamics at the output:


(7) $$x_{o,t}=x_{o,t-1}+h*(-x_{o,t-1}+p\left(\underset{j}{\Sigma }w_{j,o}x_{j,t-1}+b_{o}\right)\;\;+\beta *A\left(s\right)*\left\{a-\;x_{o,t-1}\right\}),$$

After the network reaches a clamped-phase steady state, weights could be updated according to the rule derived in the original EP paper (Scellier and Bengio, 2017):


(8) $$\Delta \,w_{p\,r\,e,p\,o\,s\,t}=\frac{1}{\beta }\,\alpha \,\left({\hat{x}}_{p\,r\,e}\,{\hat{x}}_{p\,o\,s\,t}-{\overset{ˇ}{x}}_{p\,r\,e}\,{\overset{ˇ}{x}}_{p\,o\,s\,t}\right)$$

where $\hat{x}$ is an activity at the weakly clamped phase, *x*? is an activity at the free phase, α is the learning rate, β is a nudging parameter, *pre* and *post* are previous and post layer neuron indexes, respectively (e.g., for Δ*w*_*i*,*j*_, *pre* and *post* will be *i* and *j*, respectively).

Here we replace Eq. 8 with the new rule proposed in our previous work (Luczak et al., 2022), which allows learning to occur in a single phase by assuming that neurons may predict their own future activity. The study showed that a rule of this form emerges naturally if we assume that each neuron is working to maximize its metabolic energy. The new rule is:


(9) $$\Delta \,w_{p\,r\,e,p\,o\,s\,t}\propto \frac{1}{\beta }\,\alpha \,\left({\hat{x}}_{p\,r\,e}\,{\hat{x}}_{p\,o\,s\,t}-{\hat{x}}_{p\,r\,e}\,{\overset{ˇ}{x}}_{p\,o\,s\,t}\right)\;\;=\frac{1}{\beta }\alpha {\hat{x}}_{p\,r\,e}\left({\hat{x}}_{p\,o\,s\,t}-{\overset{ˇ}{x}}_{p\,o\,s\,t}\right).$$

In this study we use this update rule for training the Actor, but omit the feature of neurons predicting their own future activity for simplicity [i.e., we assume perfect predictions by using the free-phase and clamped-phase activities directly. For details of how the prediction feature was implemented previously, see Luczak et al. (2022)]. Interestingly, this new, single-phase learning rule performs similarly or slightly better than the original rule in Eq. 8 [see Supplementary Section “Original update rule for Actor (Equilibrium Propagation)”].

### Critic network (trained by backpropagation)

Equation 5 represents a prediction error–the error between *r* + *V*(*s*′), the actual value of the present experience, and *V*(*s*), the predicted value. The mean squared prediction error is then:


(10) $$L={||\left(r+V\,\left(s^{{}^{\prime }}\right)\right)-V\,\left(s\right)||}_{2}^{2}$$

The critic network is tuned using BP in the usual way to minimize this prediction error.

### Experience replay

We use experience replay (Lin, 1992; Mnih et al., 2013, 2015; Wang et al., 2016) to make our model more stable. This method stores the agent’s experiences (including states, actions, rewards, and next-states) and makes them available for learning later. It is worth noting that experience replay is also biologically plausible; analogous to memory replay during sleep (Wilson and McNaughton, 1994).

The complete algorithm is shown in Algorithm 1.

**ALGORITHM 1:** Train Actor-Critic by EP and BP.

**Input**: Initialize action value function with synaptic weights *w* and *b.* Initialize replay memory *D*, episode size *E*, maximum step for each episode *J*. maximum iteration *T* for actor, learning rate α, nudging parameter β, time step *dt*, activation function *p*
for episode = 1, 2,…., E do
for j = 1, 2,…, J do
Compute x_j,f_ with Eqs 1, 2 // index f means free phase
Select action based on the probability of x_j,f_
Execute action a_j_ in emulator and observe reward r_j_ and state s_j__+__1_
store transition (a_j_, r_j_, s_j_, s_j__+__1_) in D
set s = s′
if D has enough transition then
Sample random minibatch of transitions (a_k_, r_k_, s_k_, s_k__+__1_) from D $$y=\left\{\begin{array}{c} r_{k},f\,o\,r\,t\,e\,r\,m\,i\,n\,a\,l\\ r_{k}+V_{\left(s_{k+1}\right)},f\,o\,r\,n\,o\,n\,t\,e\,r\,m\,i\,n\,a\,l \end{array}\right\}$$
Compute *A(_*sk*_)* with *y* and *V(_*sk*_)* by Eq. 6
// Update actor weights
Compute *x*_*k,f*_ by Eqs 1, 2 // index f means free phase
Compute *x*_*k,c*_ with *A*(s) and *a*_*k*_ by Eqs 3, 7 // index c means clamped phase
Compute *Δw* by Eq. 9 with *x_*k,f*_, x_*k,c*_*
*w ← w + Δw*
// Update critic weights
Perform a gradient stop on *_(y–V(s))^2_*
end if
end for
end for

The code to reproduce our results is located at: https://github.com/ykubo82/HybridRL.

## Experiments

**FIGURE 1:**
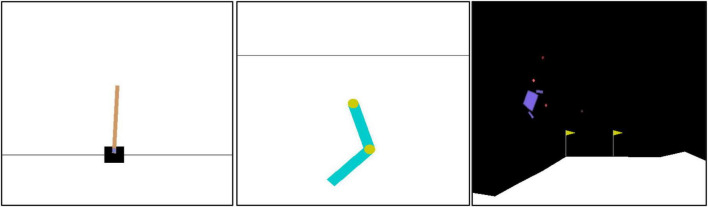
Images of environments in tasks for our model: CartPole-v0 (left), Acrobot-v1 (center), and LunarLander-v2 (right). CartPole-v0 task: A pole is on a cart, and this pole is unstable. The goal of this task is to move the cart to left or right to balance the pole. Acrobot-v1: a robot arm is composed of two joints. The goal of this task is to swing the arm to reach the black horizontal line. LunarLander-v2: There is a spaceship that tries to land. The goal of this task is to land the spaceship between the flags smoothly by moving the spaceship.

We tested our model in three simple Open AI gym tasks (Brockman et al., 2016): CartPole-v0, Acrobot-v1, and LunarLander-v2 (Figure 1 shows the images of these tasks). All of these tasks feature continuous states and discrete actions. Our model uses multilayer perceptrons for both Actor and Critic networks, trained by EP and BP, respectively (For comparison we also trained the actor using BP through time, but performance was not as good. Those results can be found in Supplementary Section “Backpropagation through time with Actor”). Each multilayer-perceptron neural network (MLP) consists of 1 hidden layer with 256 nodes. For LunarLander-v2, we increased the hidden size to 512 due to the complexity of the task.

The activation function for the hidden layer on both Actor and Critic is the hard sigmoid from Laborieux et al. (2021) for CartPole-v0 and LunarLander-v2, the hard sigmoid from Ernoult et al. (2019) for Acrobot-v1. The activation function for the Actor’s output layer is the softmax function. A maximum of 1000 experiences were stored for experience replay, and the mini-batch size was 20. The learner was allowed 1000 steps for the CartPole-v0 and Acrobot-v1 tasks, and 2000 for LunarLander-v2. Parameter settings are shown in Table 1. Experimental results for additional BP learning rates may be found in our Supplementary Section “Small learning rate for BP.” For critic networks trained by BP, we used Adam optimizer (Kingma and Ba, 2014) to accelerate models’ training.

**TABLE 1:** Parameters for our models on each task.

Task	NN Actor	NN Critic	α1 for Actor	α2 for Actor	β for Actor	α for Critic	Iteration for Actor	
							1st phase	2nd phase
CartPole	4-256-2	4-256-1	0.0001	0.0001	0.02	0.001	150	25
Acrobot	6-256-3	6-256-1	0.001	0.001	0.02	0.001	150	25
LunarLander	8-512-4	8-512-1	0.0001	0.002	0.03	0.0003	180	25

NN describes number of neurons in each layer, α_1_ is the learning rate for the weights between the input and hidden layer, α_2_ is the learning rate for the weights between the hidden and output layers, and 1st and 2nd phases mean duration of free phase and weakly clamped phases, respectively. Results for additional learning rates may be found in our Supplementary Section “The other learning rates for EP-BP”.

For comparison, we also implement a model with the same architecture as described above, but trained purely by BP. Hereafter we refer to our model with Actor trained by EP and Critic trained by backpropagation as EP-BP, while the baseline Actor-Critic model trained entirely by backpropagation as BP. All models were run eight times, and means and standard deviations were recorded.

## Results

**FIGURE 2:**
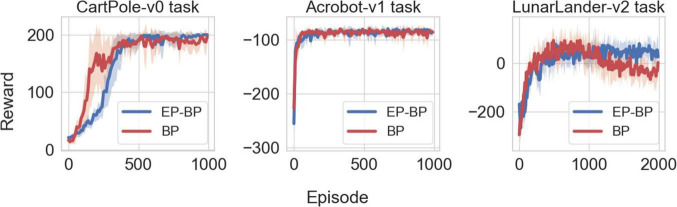
Plotting the reward vs. episode for CartPole-v0 (left), Acrobot-v1 (center), and LunarLander-v2 (right) on both backpropagation (BP) and EP-BP. Solid lines shows mean across 8 runs and shaded area denote standard deviation. Note that for Acrobot-v1, the agent receives –1 as punishment until it reaches the target.

**FIGURE 3:**
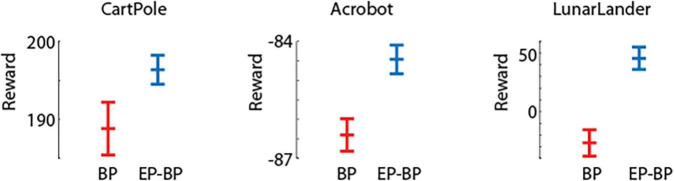
Average rewards and std error (SEM) for the last 25% of episodes for backpropagation (BP) and EP-BP on CartPole-v0, Acrobot-v1, and LunarLander-v2.

**FIGURE 4:**
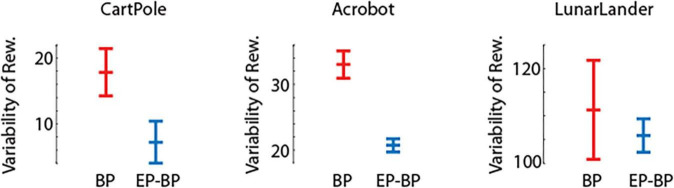
Average variability and std error (SEM) for the last 25% of episodes for backpropagation (BP) and EP-BP on CartPole-v0, Acrobot-v1, and LunarLander-v2.

Figure 2 shows performance of EP-BP and BP on each task. On all tasks our EP-BP model converges to more stable rewarding behavior than the baseline model train with BP only. This is quantified in Figure 3 which shows the mean reward obtained in the last 25% episodes. In each case the mean reward obtained by EP-BP is higher as compared to BP model. Moreover, closer examination of traces in Figure 2 showed higher variability in reward for BP trained model. To quantify it, for each of 8 runs of the model we calculated standard deviation (SD) from the last 25% of episodes. Figure 4 shows average SD across 8 runs for each model. This measure of variability was consistently lower for our EP-BP model. This tells us that our model is more stable than the base line model.

**FIGURE 5:**
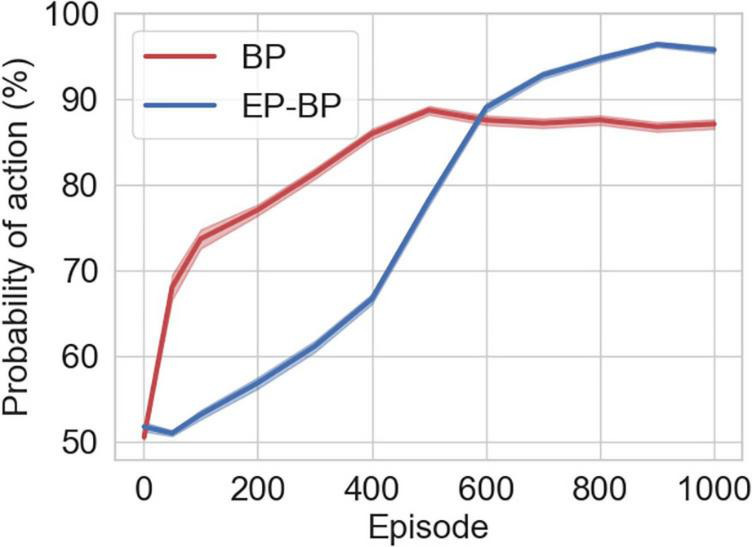
Mean and std error (SEM) (shaded area) for the probability of actions that EP-BP and backpropagation (BP) models takes on CartPole-v0.

As an internal measure of learning, similar to Römer et al. (2022), we recorded the softmax probability for each action executed by the Actor network throughout learning. For each episode, we saved the probabilities of actions that EP-BP and BP took. Figure 5 shows EP-BP executing actions with very high (>90%) confidence after about 600 episodes in the CarPole-v0 task. This means that less than 10% of actions are selected randomly. Randomness might be important for exploring the environment in the early phase for gathering information about the environment (exploration), but in the last phase, the model should take the optimal action after getting enough information (exploitation) (Maroti, 2019). However, if a model does not have a high enough confidence which action is optimal, the model might not take that action because there is still some randomness. For example, a person knows that A route is always busy with traffic jams based on his experience (thanks to exploration), thus he always takes B route to the office and arrives on time (exploitation). However, another person also knows that A route is always busy based on his experience, but he sometimes takes the A route (more often than the first person) because he does not have enough confidence for the B route (this means he thinks sometimes the B route might not be busy), and he is sometimes late. Thus, the BP model’s confidence is lower after learning, which may explain its somewhat less consistent behavior (this means the BP model takes more often non-optimal actions than EP-BP model). Of course, it is possible to drive the BP model’s action probabilities up by decreasing the temperature of the softmax operation, but this does not outperform our model (see Supplementary Section “Softmax function with low temperature for BP model”).

## Discussion

This study has explored the value of EP in reinforcement learning by proposing an Actor-Critic model with the Actor network trained by EP and the Critic network trained by BP. The resulting models learn more consistent high-reward behavior than a baseline model trained exclusively by BP. EP has been previously applied to image classification, but to our knowledge this is the first attempt to formulate an EP-based reinforcement learning system. Thus, we consider it to be an important development toward the next generation of biologically plausible algorithms. Other, future developments should include application of EP to tasks like video classification (Karpathy et al., 2014) and speech recognition (Malik et al., 2021).

By exploring an EP-BP hybrid, this work provides an important step toward a completely biologically plausible Actor-Critic model. Conventional, purely BP-based models, being not very biologically plausible, can be interpreted as purely abstract models of real biological learning processes. Here we have replaced the abstract BP-based Actor with a biologically plausible EP-based Actor, while allowing the Critic to remain an abstract model of the sophisticated neuronal system that evaluates actions. At present, we find this is necessary to achieve stability: we have implemented AC models trained purely by EP and found they do not always converge [for these results, see our Supplementary Section “Critic network (trained by Equilibrium Propagation)”]. Thus, a stable method for reducing both Actor and Critic to biologically plausible networks remains elusive.

In addition to pursuing a purely EP learning system, future work should consider a convolutional network for application to more complex tasks such as Atari games (Bellemare et al., 2013) or for neuronal data analysis tasks (Luczak et al., 2004; Luczak and Narayanan, 2005; Ponjavic-Conte et al., 2012; Ryait et al., 2019). Another avenue for exploration would be the inclusion of neural adaptation (Luczak and Kubo, 2021; Kubo et al., 2022); a biologically inspired modification to EP which previous work has shown to work well on image classification tasks, and may have value in reinforcement learning as well.

On three tasks investigated here, our EP-BP model works better than the AC trained only by BP. One of the reasons why it works better is, again, the higher probabilities of action. In the last phase of the training, we could see that our model is very stable and has higher probabilities of action. This means our model has enough information about the environment of the tasks, and the model takes optimal actions. Another, related reason may be the somewhat slower learning of EP-BP, as observed in Figures 2, 5. This could indicate a more thorough exploration of the environment in the early stages of learning.

## Data availability statement

The original contributions presented in this study are included in the article/Supplementary material, further inquiries can be directed to the corresponding authors.

## Author contributions

YK conceived the project, analyzed data, performed simulations, and wrote the manuscript. EC engaged in theoretical discussions and commented extensively on the manuscript. AL analyzed data and contributed to writing the manuscript. All authors contributed to the article and approved the submitted version.
