# Supplementary material for: Combining backpropagation with Equilibrium Propagation to improve an Actor-Critic reinforcement learning framework
Source: Front Comput Neurosci. 2022 Aug 23;16:980613. doi: 10.3389/fncom.2022.980613 (PMC9446087; doi:10.3389/fncom.2022.980613)
Supplement: Supplementary file 1 [file Data_Sheet_1.PDF]

Supplementary file for: Combining Backpropagation with Equilibrium Propagation to improve an Actor-Critic RL framework.

1. Critic network (trained by Equilibrium propagation)

We have implemented and tested an Actor-Critic trained entirely by Equilibrium propagation. The dynamics for Actor are calculated by Equations 3 and 4, and Critic is trained by Equation 3 and 11 as follows:

The error between $r + V(s')$, the actual value of the present experience, and $V(s)$, the predicted value. The mean squared prediction error with Equilibrium propagation is then:

$$x_{o,t} = x_{o,t-1} + h * (- x_{o,t-1} + p\left(\sum_j w_{j,o} x_{j,t-1} + b_o\right) + \beta * (r + V(s') - x_{o,t-1}) \quad \text{(S1)}$$

Results:

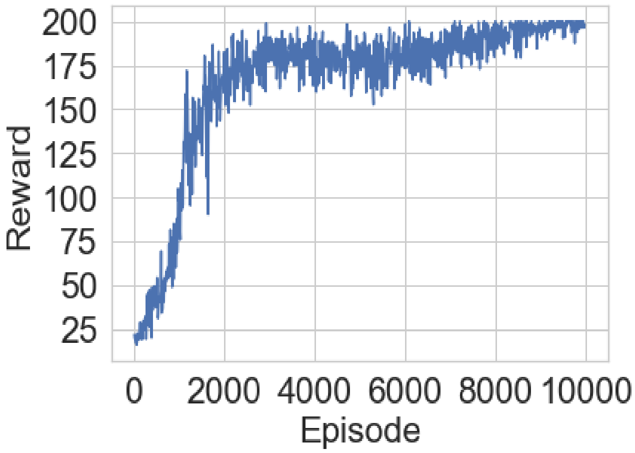

Figure S1 (Supplemental): Plotting the reward vs episode for CartPole-v0 on EP. Figure S1 shows that one of the results for AC trained by only EP. However, for some runs of the same algorithm network did not achieve maximum reward. The hyper-parameters used for those simulations are listed as below:

We trained AC trained by only EP (EP) with the hyper-parameters as below:

Table S1 (Supplemental): One of example parameters for our models trained by only EP on CartPole. NN describes number of neurons in each layer, α_1 is the learning rate for the weights between the input and hidden layer, α_2 is the learning rate for the weights between the hidden and output layers, and steps for 1st and 2nd phases are the same as EP. Furthermore, we set up longer episodes for this training (from 1000 to 10000).

Task	NN Actor	NN Critic	α1 for Actor	α2 for Actor	β for Actor	α1 for Critic	α2 for Critic	β for Critic
CartPole	4-256-2	4-256-1	0.0001	0.0001	0.03	0.001	0.008	0.01

At present, a stable method for reducing both Actor and Critic to biologically plausible networks (trained by EP) remains elusive.

2. Dynamics for Actor:

We did not explain how our dynamics for Equations 5 and 7 come from. Here we would like to write the explanation for Equations.

In our manuscript, we describe Equation 5 as:

$$x_{o,t} = x_{o,t-1} + h * (- x_{o,t-1} + p\left(\sum_j w_{j,o} x_{j,t-1} + b_o\right) + \beta * V * (a - x_{o,t-1})), \tag{S2}$$

and Equation 7 as:

$$x_{o,t} = x_{o,t-1} + h * (- x_{o,t-1} + p\left(\sum_j w_{j,o} x_{j,t-1} + b_o\right)$$

$$+\beta * A * (a - \ x_{o,t-1})). \quad \text{(S3)}$$

Original dynamics for Equilibrium propagation is:

$$x_{o,t} = x_{o,t-1} \ + \ h * (- x_{o,t-1} + p\left(\sum_j w_{j,o} x_{j,t-1} \ + b_o\right)$$

$$+\beta * (y - \ x_{o,t-1})), \quad \text{(S4)}$$

where y is a target. The last part of this equation is the loss function, and this part nudges the activation to minimize the target and activation. On the other hand, in Eqs S2 and S3, the last part of this equation or nudging part is based on log probability of actions $\sum_i A * log(a_i|x_i)$ (the derivative of this is $\sum_i A * (a_i - \ x_i)$ with respect to the weights. This is based on *Policy Gradients*) where *a* is an action that a model takes, *x* is the probability of the actions for the model, and *i* is a sample index. The goal of learning is to increase the probability of the actions that work and decrease the probability of the actions that do not. To achieve this, the difference between *a* (actual action) and *x* (action probability) is scaled by *V* or *A*, which will be positive for rewarding actions and negative for actions that bring negative reward. Kapathy [1] discuss that this will maximize the log probability of actions that lead to good outcome and minimize the log probability that do not. Thus, Eqs S2 and S3, (especially the last terms) nudge the activations to increase the probability for good outcome but minimize the probability for bad outcome.

3. The other learning rates for EP-BP:

We have tried several learning rates for our model on each task. Even though we change the leaning rates, we still get a good performance. For example, when we increased the learning rates for actor to 1e-3 and 1e-3 from 1e-4 and 1e-4, we still get a similar result to the original parameter settings on CartPole-v0 (Figure S2 - left). Furthermore, when we reduced the learning rates for actor to 1e-4 and 1e-4 from 1e-3 and 1e-3 on Acrobot-v1, we still get a similar result to the original parameter settings (Figure S2 - center).

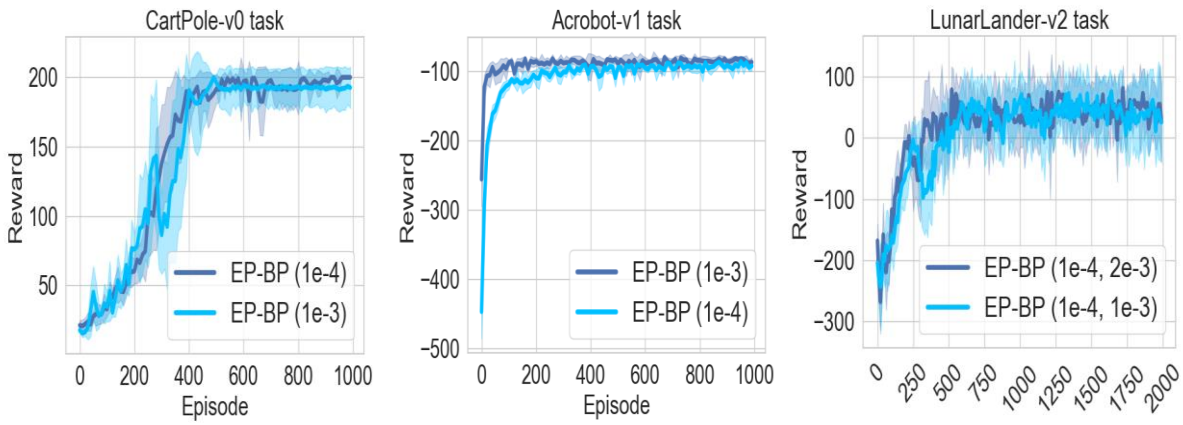

Figure S2 (Supplemental): Plotting the reward vs episode for CartPole-v0 (left), Acrobot-v1 (center), and LunarLander-v2 (right) on EP-BP. Blue line is our best model (the model shown in our manuscript), and red line is our model with the changed learning rates for Actor (1e-3 and 1e-3 from 1e-4 and 1e-4 for CartPole-v0, 1e-4 and 1e-4 from 1e-3 and 1e-3 for Acrobot-v1, 1-e4 and 1e-3 from 1-e4 and 2e-3 for LunarLander-v2).

4. Original update rule for Actor (Equilibrium propagation):

We used our update rule for our model on all the task:

$$\Delta w_{pre,post} \propto \frac{1}{\beta}\alpha(\hat{x}_{pre}\hat{x}_{post} - \hat{x}_{pre}\check{x}_{post}) = \frac{1}{\beta}\alpha\, \hat{x}_{pre}(\hat{x}_{post} - \check{x}_{post}). \quad \text{(S5)}$$

However, we still do not know if our update rule is better than the original update rule or not.

The original update rule is:

$$\Delta w_{pre,post} = \frac{1}{\beta}\alpha\left(\hat{x}_{pre}\hat{x}_{post} - \check{x}_{pre}\check{x}_{post}\right). \quad \text{(S6)}$$

We compare these update rules on all the tasks. Hyper-parameters are all the same as previous experiments.

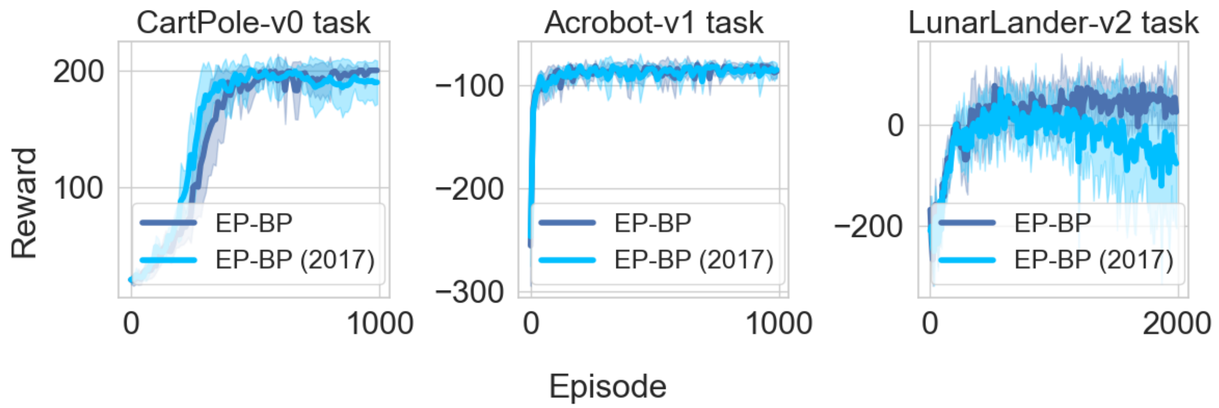

Figure S3 (Supplemental): Plotting the reward vs episode for CartPole-v0 (left), Acrobot-v1 (center), and LunarLander-v2 (right) on both EP-BP and EP-BP (2017), which uses the update rule Eq 3, originally introduced in 2017 by Scellier et al [2]. Solid lines shows mean across 8 runs and shaded area denote standard deviation. Note that for Acrobot-v1, the agent receives -1 as punishment until it reaches the target.

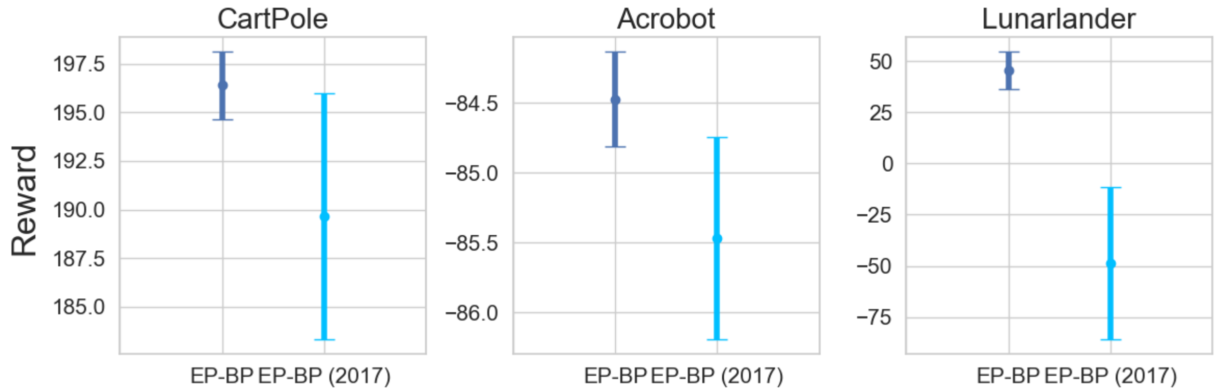

Figure S4 (Supplemental): Average rewards and std error (SEM) for the last 25% of episodes for EP-BP and EP-BP (2017), which uses the update on CartPole-v0, Acrobot-v1, and LunarLander-v2.

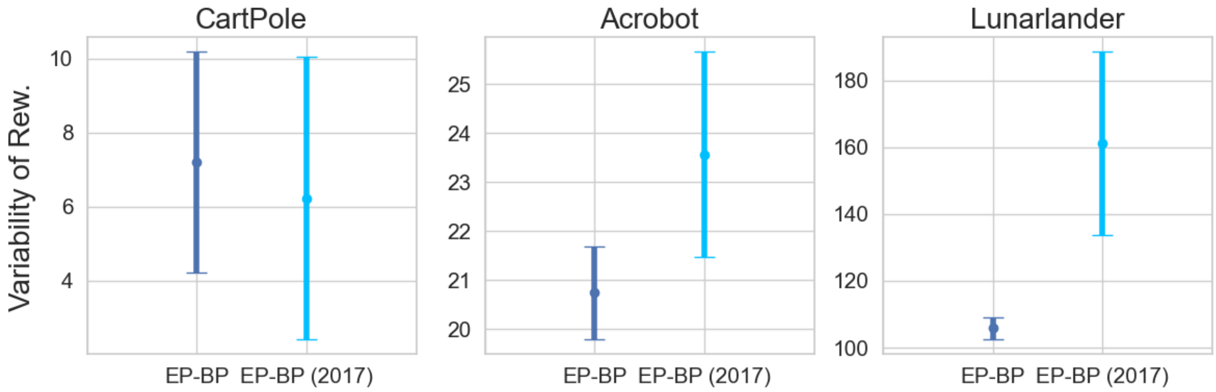

Figure S5 (Supplemental): Average variability and std error (SEM) for the last 25% of episodes for EP-BP and EP-BP (2017), which uses the update on CartPole-v0, Acrobot-v1, and LunarLander-v2.

Figures S3, S4, and S5 shows that our update rule achieves higher final reward than the original update rule, especially on LunarLander-v2. EP-BP with the original update rule learns faster on the CartPole-v0 task, but the updated rule seems to converge to a slightly better solution in the end.

5. Softmax function with low temperature for BP model:

In our manuscript, we suggest that our model is better than the backpropagation (BP) model because of the high action probabilities (i.e. high confidence in actions) observed during tasks. Of course, it is possible to increase action probability by decreasing the temperature of the softmax operation at the network output. Here, we run BP models with low softmax temperatures (0.01) for the last 25% of the episodes on each task. Hyper-parameters are left the same as in previous experiments.

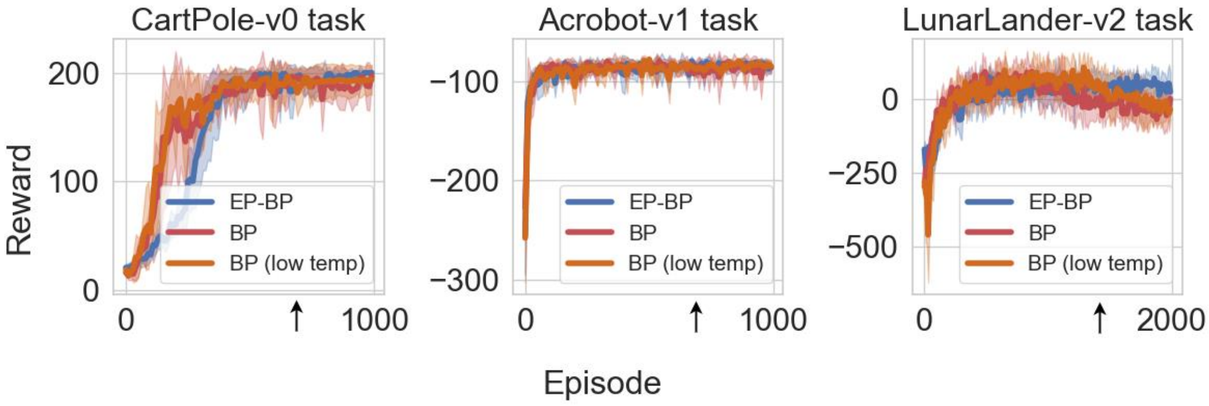

Figure S6 (Supplemental): Plotting the reward vs episode for CartPole-v0 (left), Acrobot-v1 (center), and LunarLander-v2 (right) on EP-BP, BP, and BP (low temp), which uses softmax temperature = 0.01 in the last 25% of episodes. Solid lines show mean across 8 runs and shaded area denote standard deviation. Note that for Acrobot-v1, the agent receives -1 as punishment until it reaches the target. The arrows are for starting points of lower softmax temperature.

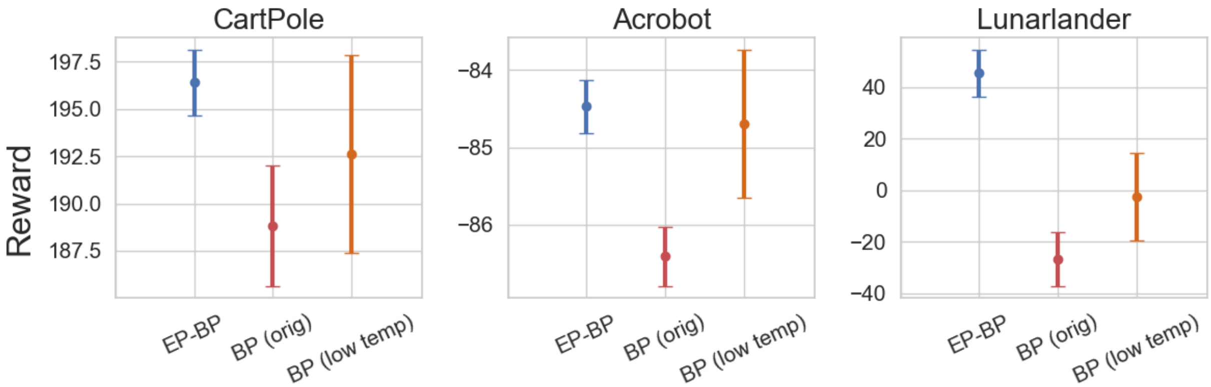

Figure S7 (Supplemental): Average rewards and std error (SEM) for the last 25% of episodes for EP-BP, BP, and BP (low temp), which uses softmax temperature = 0.01 in the last 25% of episodes.

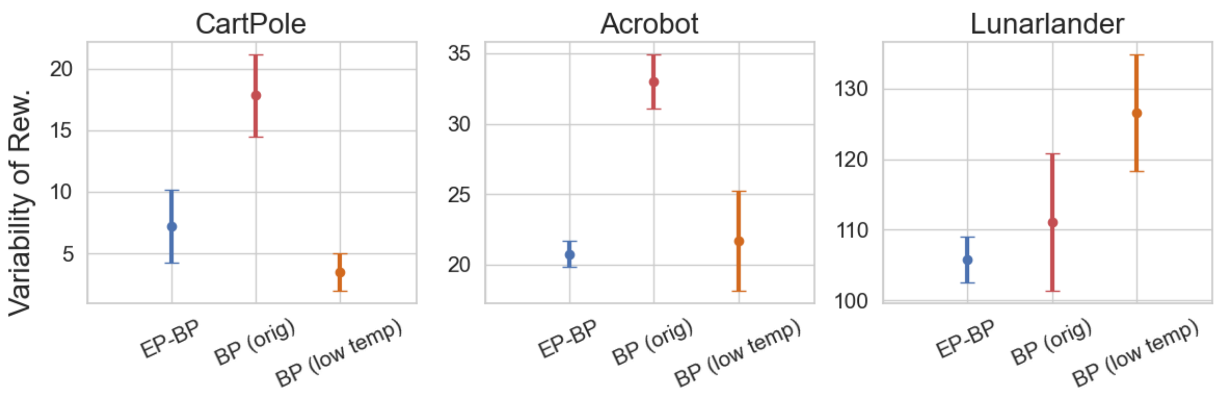

Figure S8 (Supplemental): Average variability and std error (SEM) for the last 25% of episodes for EP-BP, BP, and BP (low temp), which uses softmax temperature = 0.01 in the last 25% of episodes.

Figures S6, S7, S8 show that decreasing the softmax temperature in this way has an effect on the BP model's performance, and BP (low temp) outperforms the original BP. However, it still does not outperform the EP-BP model but is more competitive than the original BP. On the LunarLander-v2 task, we also tested decreasing the BP softmax temperature earlier than the last 25% episodes (specifically, after 750 episodes). In that case, the reduced temperature makes a more noticeable difference in performance, but still does not outperform the EP-BP model.

6. Small learning rate for BP:

Since BP increases its reward faster and we mentioned the speed of learning might be a factor for EP-BP to achieve better outcomes, we changed the learning rate for BP to the smaller learning rate.

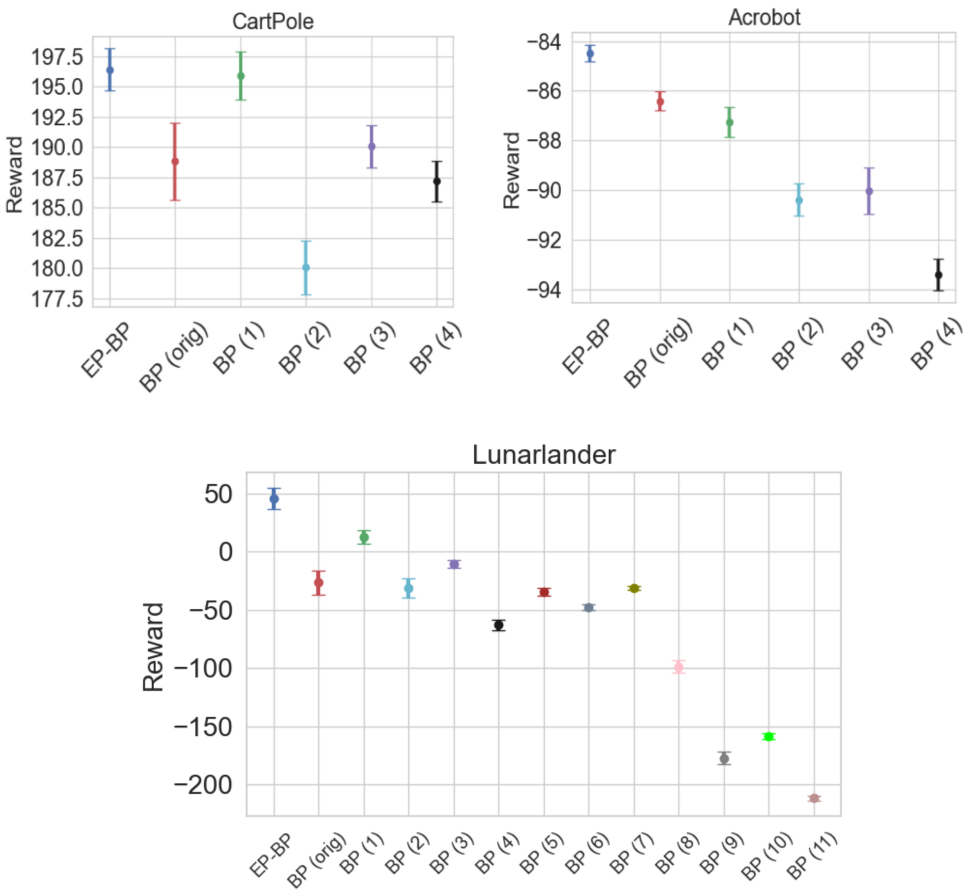

Figure S9 (Supplemental): Average rewards and std error (SEM) for the last 25% of episodes for EP-BP and BPs on CartPole-v0, Acrobot-v1, and LunarLander-v2. BP (orig) is the original leaning rates in our manuscript. BP with numbers are BP with the changed learning rates. (BP(x) refers to runs with different hyperparameters. As described in Table S2 below)

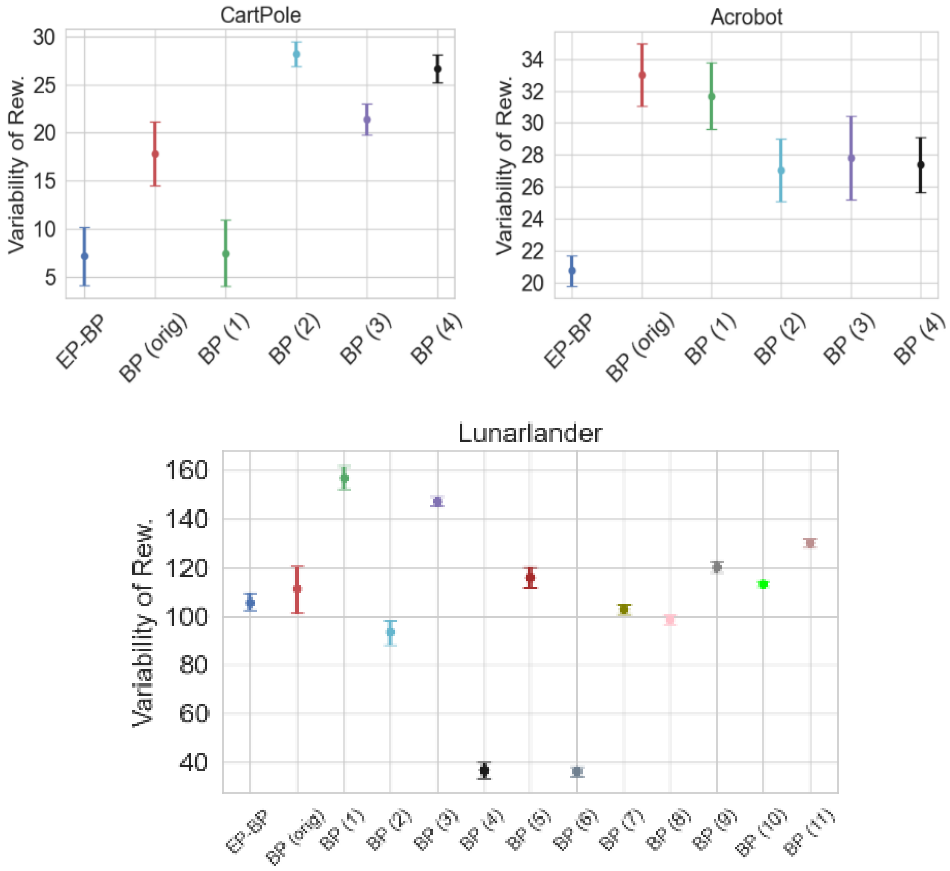

Figure S10 (Supplemental): Average variability and std error (SEM) for the last 25% of episodes for BP and EP-BP on CartPole-v0, Acrobot-v1, and LunarLander-v2.

Table S2 (Supplemental): Parameters for our models trained by BP on CartPole-v0, Acrobot-v1, and LunarLander-v2. NN describes number of neurons in each layer, α is the learning rate. Original means the learning rates in our manuscript. The numbers with parentheses are the number for Figure S9 (For example, BP (1) in Figure S9 uses the learning rate = 1-e3 and 1-e3 for actor and critic respectively).

CartPole-v0	α for actor	α for critic	Acrobot-v2	α for actor	α for critic	LunarLander-v2	α for actor	α for critic
original	2e-3	1e-3	original	2e-3	1e-3	original	2e-3	1e-4
(1)	1e-3	1e-3	(1)	1e-3	1e-3	(1)	2e-3	1e-5
(2)	2e-4	1e-3	(2)	2e-4	1e-3	(2)	1e-3	1e-4
(3)	2e-4	1e-4	(3)	2e-4	1e-4	(3)	1e-3	1e-5
(4)	1e-4	1e-4	(4)	1e-4	1e-4	(4)	2e-4	1e-4
						(5)	2e-4	1e-5
						(6)	1e-4	1e-4
						(7)	1e-4	1e-5
						(8)	2e-5	1e-4
						(9)	2e-5	1e-5
						(10)	1e-5	1e-4
						(11)	1e-5	1e-5

Figure S9 shows the mean reward obtained in the last 25% episodes. In each case the mean reward obtained by EP-BP is still higher as compared to BP model even though BP model with changed learning rates increased some performance on tasks, especially, CartPole-v0 and LunerLandar-v2 (BP (1)). Figure S10 shows average SD across 8 runs for each model. On LunerLandar-v2, this measure of variability was not consistently lowest for our EP-BP model, but it is still consistently lower for our model on CartPole-v0 and Acrobot-v2

7. Backpropagation through time with Actor:

To train a recurrent neural network, most of researchers use Backpropagation through time (BPTT). In this section, we trained Actor by BPTT (BPTT-BP) instead of Equilibrium

propagation. We briefly tested this model on CartPole-v0 task, which is the simplest task among the experiments. This implementation for BPTT is based on Ernoult et al [3].

Table S3 (Supplemental): One of example parameters for our models trained by BPTT-BP on CartPole. NN describes number of neurons in each layer, α_1 is the learning rate for the weights between the input and hidden layer, α_2 is the learning rate for the weights between the hidden and output layers, and steps for 1st phase is 180. We did not use any optimizer for Actor such as Adam [4], but used it for Critic.

Task	NN Actor	NN Critic	α1 for Actor	α2 for Actor	α for Critic
CartPole	4-256-2	4-256-1	0.0001	0.001	0.001

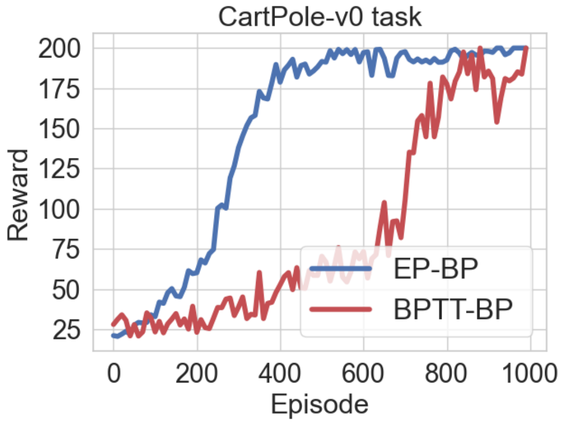

Figure S11 (Supplemental): Plotting the reward vs episode for CartPole-v0 on EP-BP and BPTT-BP whose actor is trained by BPTT. the performances of EP and BPTT-BP (best performance) are compared.

Figure S11 shows that EP-BP is better and more stable than BPTT-BP even though it is the best performance for BPTT-BP that is very slow to learn the environment. However, for some runs of the same algorithm network for BPTT-BP did not achieve maximum reward. The hyper-parameters used for those simulations are listed in Table S3.

References:

1. Karpathy, A. *Deep Reinforcement Learning: Pong from Pixels*. 2016; Available from: http://karpathy.github.io/2016/05/31/rl/.
2. Scellier, B. and Y. Bengio, *Equilibrium propagation: Bridging the gap between energy-based models and backpropagation.* Frontiers in computational neuroscience, 2017. **11**: p. 24.
3. Ernoult, M., et al., *Updates of equilibrium prop match gradients of backprop through time in an RNN with static input.* Advances in neural information processing systems, 2019. **32**.
4. Kingma, D.P. and J. Ba, *Adam: A method for stochastic optimization.* arXiv preprint arXiv:1412.6980, 2014.
